# Localisation of Epithelial Cells Capable of Holoclone Formation *In Vitro* and Direct Interaction with Stromal Cells in the Native Human Limbal Crypt

**DOI:** 10.1371/journal.pone.0094283

**Published:** 2014-04-08

**Authors:** Marc A. Dziasko, Hannah E. Armer, Hannah J. Levis, Alex J. Shortt, Stephen Tuft, Julie T. Daniels

**Affiliations:** 1 Department of Ocular Biology and Therapeutics, UCL Institute of Ophthalmology, London, United Kingdom; 2 Imaging unit, UCL Institute of Ophthalmology, London, United Kingdom; 3 Moorfields Eye Hospital NHS Foundation Trust, London, United Kingdom; University of Montréal and Hôpital Maisonneuve-Rosemont, Canada

## Abstract

Limbal epithelial stem cells (LESCs) are essential to maintain the transparent ocular surface required for vision. Despite great advances in our understanding of ocular stem cell biology over the last decade, the exact location of the LESC niche remains unclear. In the present study we have used *in vitro* clonal analysis to confirm that limbal crypts provide a niche for the resident LESCs. We have used high-resolution imaging of the basal epithelial layer at the limbus to identify cells with a morphology consistent with stem cells that were only present within the basal layer of the limbal crypts. These cells are proximal to limbal stromal cells suggesting direct cell-to-cell interaction. Serial block-face scanning electron microscopy (SBFSEM) confirmed that the putative LESCs are indeed in direct contact with cells in the underlying stroma, a contact that is facilitated by focal basement membrane interruptions. Limbal mesenchymal cells previously identified in the human limbus collocate in the crypt-rich limbal stromal area in the vicinity of LESCs and may be involved in the cell-to-cell contact revealed by SBFSEM. We also observed a high population of melanocytes within the basal layer of the limbal crypts. From these observations we present a three dimensional reconstruction of the LESC niche in which the stem cell is closely associated and maintained by both dendritic pigmented limbal melanocytes and elongated limbal stromal cells.

## Introduction

The cornea, the transparent tissue located at the front of the eye, is a highly specialized tissue that transmits and refracts light onto the retina. The outer layer of the central cornea over the visual axis is composed of a stratified squamous epithelium that is continuously replaced from a population of epithelial stem cells, as is the case with the epidermis, the hair follicle and the epithelium of the small intestine [1], [2].

Currently, the prevailing hypothesis is that in most species, stem cells of the ocular surface are located in the basal layer of the epithelium at the limbus, which is the 1.5–2 mm wide interface between the peripheral avascular cornea and adjacent conjunctiva [3], [4]. Radial ridges of the underlying stromal (palisades) at the superior and inferior limbus are associated with LESC rich areas. It is widely accepted that LESCs are the smallest cells in the basal layer with a high nucleus-cytoplasm ratio [5], and that they express a panel of putative stem cell markers including the transporter ABCG2 [6], transcription factors such as p63 [7] and its ΔNp63α isoform [8], [9], cell adhesion molecules such as integrin α9 [6] and N-cadherin [10] and that they have a high proliferative potential in culture [11], [12].

LESCs are maintained and concentrated in a stem cell niche where they have the ability to self renew and to preserve their multipotency [3]. The elements of this microenvironment consists of soluble factors, cell-to-cell interactions between the other cells in the niche and a unique composition of the local extracellular matrix [13]–[16]. In the last decade, specific anatomical features have been described within the human limbus. Serial histological sectioning revealed ‘distinct anatomical extensions from the peripheral aspect of the limbal palisades’. These were termed ‘limbal epithelial crypts’ and were proposed as a putative LESC niche owing to the presence of cells expressing putative stem cell markers including ABCG2 [17]. In 2007, Shortt et al. described epithelial cell filled crypts between the limbal palisades of Vogt corresponding to the ‘interpalisadal epithelial rete ridges’ described by Goldberg and Bron [18]. These were termed ‘limbal crypts’ (LCs). The LC, similar in structure to the rete pegs of the epidermis, also expressed high levels of putative LESCs markers as determined by examination of whole-mounted tissue using immunochemical confocal microscopy. Furthermore, the epithelial cells isolated from LCs presented high colony forming efficiency potential *in vitro* – the first functional evidence attributed to either of the proposed anatomical niche structures [19]. Hence LCs were proposed as a putative candidate for the LESC niche. However at that time, the gold standard single cell clonal analysis assay, used to identify epithelial stem cells cultured *in vitro* [20], was not performed causing ambiguity regarding the relevance of LCs as a stem cell niche. Interestingly, LCs that were more often observed at the superior and inferior human limbus could not be detected in patients affected by LESC deficiency [19].

As reported in other tissues [21], [22], an important feature of a stem cell niche is interaction between stem cells and their surrounding niche cells. Stromal cells were previously observed in close proximity to putative LESCs lining LCs of the human limbus [19]. A close spatial relationship involving cytokine cellular-crosstalk between basal epithelial cells and limbal stromal cells was also discovered in the LC [15]. The emerging concept from different studies suggests that LESC may be maintained by direct contact with their niche cells and that this physical interaction appears to be essential, at least *in vitro,* for maintenance of the stem cell phenotype [23]–[25].

The aims of this study were to demonstrate the previously identified LCs as a LESC niche by using functional *in vitro* clonal analysis of resident cells and, secondly, to use state-of-the art imaging techniques to observe putative LESCs in their native environment to highlight potential physical cell-to-cell interactions with surrounding niche cells.

## Materials and Methods

### 1. Ethics Statement

All human tissue was handled according to the tenets of the Declaration of Helsinki and written consent was acquired from next of kin of all deceased donors regarding eye donation for research. Research consent was obtained via the Moorfields Eye Hospital Lions Eye Bank (U.K) http://www.moorfields.nhs.uk/Aboutus/Clinicalsupportservices/Eyebank and Lions Eye Institute (Florida, U.S) http://www.fleb.org/. All experiments were approved by the National Research Ethics Service, South west 3 REC, reference 10/H0106/57.

### 2. Isolation and Culture of Limbal Epithelial Cells

Limbal palisades, which surround the LCs were macroscopically identified under a dissecting microscope. After dissection, crypt-rich (from superior and inferior limbus) and non-crypt rich (from temporal and nasal limbus) limbal biopsies isolated from the same donor were transferred separately into a solution containing 1.2 U/mL dispase II (Roche diagnostics GmbH, Mannheim, Germany) in corneal epithelial cell culture medium (CECM) containing a 1∶1 ratio of DMEM:F12, 10% (v/v) fetal bovine serum, 100 U/mL penicillin, 100 μg/mL streptomycin, 0.25 μg/mL Fungizone, epidermal growth factor (EGF) 10 ng/mL (Life technologies, Paisley, UK), hydrocortisone (0.4 μg/mL), insulin (5 μg/mL), adenine (0.18 mM), transferrin (5 μg/mL), T3 (2 nM), cholera toxin (0.1 nM) (Sigma-Aldrich, Dorset, UK) and incubated for 2 hours at 37°C. Limbal epithelial cells (LECs) were isolated from the crypt-rich and non-crypt rich biopsies by gently scraping the epithelium using the point of thin forceps. LECs were then pre-expanded for 7 days in T25 flasks on a 3T3 feeder layer that had been previously growth arrested with 4 μg/mL mitomycin C (Sigma-Aldrich, Dorset, UK) for 2 hours. CECM culture medium was changed three times a week and the co-cultures maintained at 37°C in a humidified atmosphere containing 5% CO_2_ in air.

### 3. Clonal Analysis

Cadaveric human corneas were obtained from three donors (age range 51–71 years; mean 58.3 years). In total, 124 clones isolated from the crypt-rich and non-crypt limbal biopsies from three donors were analyzed for their *in vitro* growth potential. When they had reached 50% confluence in primary 3T3-LECs co-cultures, the LECs were isolated using serial trypsinization. The 3T3 feeder cells were detached and removed using 0.05% trypsin-0,02% EDTA, before 0.5% trypsin-0.2% EDTA (Life technologies, Paisley, UK) was used to detach and prepare a single cell suspension of LECs. For each donor, 250 single LECs initially isolated from crypt-rich or from non-crypt limbal biopsies were seeded onto 55 mm^2^ plates containing 1.4×10^6^ growth-arrested 3T3s. Plates were then checked under an inverted microscope to confirm seeding of a single LECs suspension on top of growth arrested 3T3s. After 7 days in culture, small colonies (clones) of approximately 1 mm diameter were randomly selected and isolated using 8 mm sterile cloning cylinders (Sigma-Aldrich, Dorset, UK) and 0.5% trypsin-0.2% EDTA. Limbal epithelial cells from these single cell colonies (clone) were again seeded onto a 55 mm^2^ plate containing 1.4×10^6^ growth-arrested 3T3s. Epithelial cells were expanded for up to 12 days and plates fixed with 4% paraformaldehyde (PFA) (VWR International Ltd. Leicestershire, UK) and stained with 2% rhodamine. Each plate was then scored as holoclone, meroclone or paraclone depending on the percentage of aborted colonies [20]. When 0–5% of the total colonies were terminally differentiated, the clone was scored as a holoclone. When more than 95% of colonies were terminally differentiated, the clone was scored as a paraclone. Finally, when >5% but <95% of colonies were terminally differentiated, the clone was classified as a meroclone.

### 4. Transmission Electron Microscopy

Biopsies from crypt-rich and non-crypt rich regions of the human limbus were fixed in 2.5% glutaraldehyde and 2% paraformaldehyde in 0.08 M sodium cacodylate buffered to pH 7.4. Limbal biopsies were then post-fixed with 1% aqueous osmium tetroxide (Elektron Technology Ltd. Essex, UK) for 2 hours at 4**°**C and rinsed in distilled water. Following osmication, the samples were dehydrated through an ascending ethanol series (50%, 70%, 90% and 100%), passed through propylene oxide and infiltrated with 50∶50 propylene oxide: epoxy araldite resin mixture (Elektron Technology Ltd. Essex, UK) before being embedded in full resin at 60**°**C overnight. 75 nm ultra-thin sections were collected on copper grids and post-stained with lead citrate, prior to examination in a JEOL 1010 transmission electron microscope and imaged with an SC1000 Orius CCD camera (Gatan, Abingdon Oxon, UK).

### 5. Serial Block-face Scanning Electron Microscopy (SBFSEM)

Crypt-rich human limbal biopsies were fixed in 2.5% glutaraldehyde and 2% paraformaldehyde in 0.08 M sodium cacodylate buffered to pH 7.4. Tissues were washed in cold cacodylate buffer containing 2 mM calcium chloride and incubated in a solution containing an equal volume of 2% aqueous osmium tetroxide and 3% potassium ferrocyanide in 0.3 M cacodylate buffer with 4 mM calcium chloride. Tissues were then washed with double distilled water (ddH_2_0) and placed in a freshly prepared and filtered thiocarbohydrazide solution (0.01 g/mL in ddH_2_0). After being rinsed with ddH2O, tissues were placed in 2% osmium tetroxide in ddH_2_O for 30 minutes at room temperature, washed in ddH_2_O and placed in 1% uranyl acetate overnight at 4**°**C. After a rinse with ddH_2_0, tissues were placed in freshly prepared Walton’s lead aspartate solution and placed in a 60°C oven for 30 minutes. Tissues were then washed with ddH_2_0 and dehydrated through increasing concentrations of ethanol (20%, 50%, 70%, 90% and 100%). After dehydration, tissues were transferred to acetone before being infiltrated in mixtures of resin:acetone 25%, 50%, 75% respectively. Tissues were placed in 100% resin (Taab hard-plus resin, TAAB Laboratories Equipment Ltd) for 2 hours before being embedded in a fresh resin at 60°C for 48 hours. Resin blocks containing the tissues were mounted on cryo-specimen pins (Leica Microsystems, Milton Keynes, UK), trimmed and sputter-coated with a layer of gold/palladium. Samples were observed using a Zeiss SIGMA VP field emission scanning electron microscope. Serial block-face (SBF) imaging used the 3View system (Gatan, Abingdon Oxon, UK) with a low kV backscattered electron detector. This technique involves the sequential removal of the surface of the resin block using an ultra-microtome inside the microscope chamber. Ultrathin sections (100 nm) are serially cut from the resin block followed by imaging of the freshly exposed block surface. This procedure was repeated up to 1000 times generating a large data set and allowing a complete three-dimensional reconstruction of the tissues imaged.

### 6. Manual Segmentation and 3D Reconstruction

SBF scanning electron microscopy datasets were converted into voxels (volumetric picture elements) and areas of interest manually segmented and reconstructed into a 3D volume using Amira (Visage Imaging Inc.).

### 7. Immunohistochemistry

Limbal biopsies from human corneas (n = 3) were isolated under a dissecting microscope and small pieces of tissue were embedded in OCT compound. Thick frozen sections (7 μm) were cut from the crypt rich and non-crypt rich limbus tangential to the corneal circumference on a cryostat and mounted on superfrost plus microscope slides (VWR International, West Sussex, UK). Frozen sections were fixed with 4% PFA, permeabilized with 0.5% Triton-X and blocked with 10% goat serum in PBS at room temperature for 90 minutes. Sections were then incubated with primary antibodies against CD90 (Abcam ab23894, Cambridge, UK), CD105 (Abcam ab44967, Cambridge, UK) and MelanA (Abcam ab51061, Cambridge, UK) at 4°C overnight. Sections were washed and incubated with secondary antibody (Alexa-594 conjugated goat anti-mouse, 1∶250, Invitrogen A11032 for CD90 and CD105 and Alexa-594 conjugated goat anti-rabbit, 1∶250, Invitrogen A11037, Life technologies, Paisley, UK) for 1 hour at room temperature and mounted in Vectashield with DAPI (Vector laboratories Ltd. Peterborough, UK). Images were captured using a Zeiss 710 confocal microscope (Carl Zeiss, Hertfordshire, UK).

### 8. Histological Staining of Cryosections

Thick frozen sections (7 μm) tangential to corneal circumference from crypt rich and non-crypt rich limbal biopsies were fixed in 4% PFA before being stained with haematoxylin and eosin and mounted in DPX. Sections were imaged using a Nikon Eclipse TS100 inverted microscope.

### 9. Statistical Analysis

Fisher’s exact test has been used to show that the frequencies of holoclones, meroclones and paraclones observed differ significantly between the limbal crypts and the non-crypt rich limbus.

## Results

### 1. LCs Provide an Epithelial Stem Cell Niche Demonstrated by Clonal Analysis of Resident Cells

**Figure 1:**
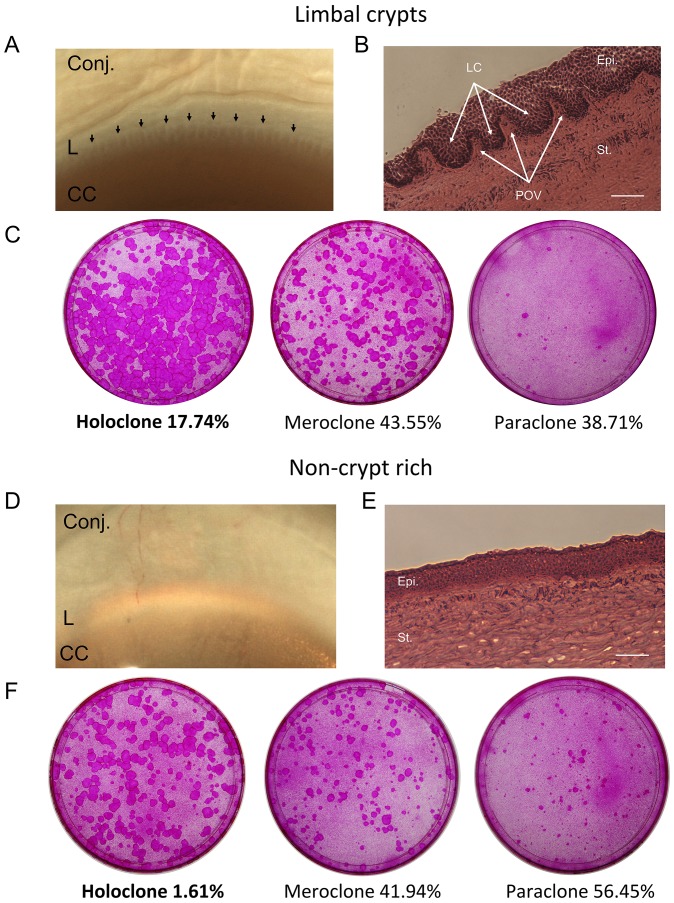
Clonal analysis. Crypt-rich (A) and non-crypt rich (D) limbal biopsies observed under the dissecting microscope and targeted for clonal analysis. Sections were cut tangentially to the limbal circumference and stained with hematoxylin and eosin. Sections shown highlight the palisades of Vogt and the limbal crypts (B) and the non-crypt rich limbus (E). Colonies of limbal epithelial cells grown in Petri dishes and stained with 2% rhodamine (C) and (F). Growth potential of single epithelial cells isolated from crypt-rich (C) and non-crypt rich (F) human limbal biopsies was characterized by the generation of holoclones, meroclones and paraclones. LECs isolated from the limbal crypts generated the highest proportion of holoclones demonstrating their stem characteristics and the LCs as a stem cell niche. Scale bars: 50 μm. Black arrows: Limbal crypts. Conj.: conjunctiva; L: Limbus; CC: central cornea; Epi.: epithelium; St.: stroma; POV: palisades of Vogt; LC: limbal crypt.

LCs were identified under a dissecting microscope (Fig. 1A) and appear as downward projections of the limbal epithelium into the limbal stroma enclosed laterally by the limbal palisades – the LC-rich limbus (Fig. 1B). Where these structures were absent, the region was termed non-crypt rich limbus as shown in Figure 1D and E. Among three human donors and 124 clones analyzed, cells isolated from the LC were able to generate the highest proportion of holoclones (17.74%, 11 holoclones among 62 clones) compared to LECs isolated from the non crypt-rich limbus in which only one holoclone was observed among 62 clones analyzed (1.61%). Cells isolated from the non-crypt rich limbus showed a lower growth potential when compared to those isolated from the crypt-rich limbus (56.45% paraclones cf. 38.71% paraclones respectively). The number of meroclones (43.55% for cells isolated from the LCs and 41.94% from the non-crypt rich) was similar for both limbal areas (Fig. 1C, F and table 1).

**Table 1:** Clonal analysis.

Origin of tissue	Number of donors	Age of donors	Number of holoclones	Number of meroclones	Number of paraclones	Total
***Limbal crypts***	3	51–71	11	27	24	62
***Non-crypt rich***	3	51–71	1	26	35	62
***P-value***	p = 0.0038*					

Single limbal epithelial cells were isolated from 6 primary co-cultures originated from crypt-rich and non-crypt rich limbal biopsies of three human donors. After 7 days, single clones were isolated and transferred to a new culture dish and expanded for 12 days prior to fixation and rhodamine staining. Clones were finally classified as holoclones, meroclones or paraclones depending on the percentage of aborted colonies**.** *is considered as statistically significant (Fisher’s exact test p<0.005).

### 2. LCs Support a Subpopulation of LECs Exhibiting Stem Cell Morphology that are Closely Associated with the Underlying Stromal Cells

**Figure 2:**
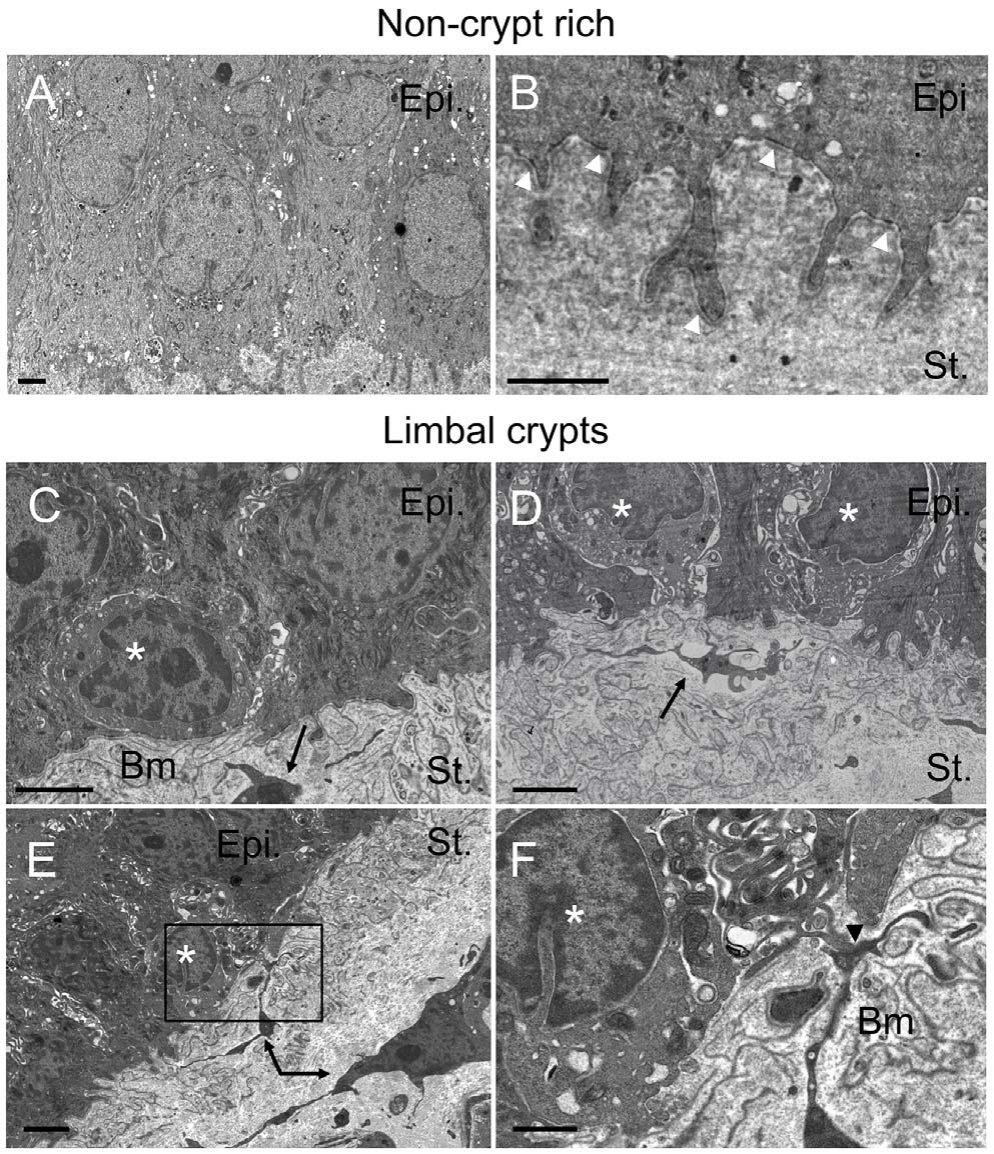
Observation of putative LESCs by TEM within the LCs. Transmission electron micrographs highlighting the interface between the limbal stroma and the limbal basal epithelial layer within the non-crypt rich limbus (A, B) and the limbal crypts (C, D, E, F). White arrowheads: hemidesmosomes; Black arrows: limbal stromal cell extensions in the vicinity of putative LESCs. Epi.: epithelium; St.: stroma; Bm: Basement membrane. White asterisks: putative LESCs. Black arrowhead: Contact between putative LESC and limbal stromal cell. Black box in E represents area in F. Scale bars A, B, C, D and E: 2 μm, F: 1 μm.

In the non-crypt rich limbus, transmission electron micrographs show a uniform limbal basal epithelial cell population (Fig. 2A). These epithelial cells appeared columnar and elongated, expressed intermediate filaments and had basal finger-like projections with an abundance of hemidesmosomes (Fig. 2B).

In contrast, the limbal basal epithelial layer in the crypt-rich limbus contained a mixed population of epithelial cells. Most of the cells had the same morphological characteristics as the basal epithelial layer of the non-crypt rich limbus. However, we also observed a population of small, circular, basal cells characterized by a high nucleus/cytoplasm ratio that were mainly located on the edges of the crypt close to blood vessels in the underlying stroma. These cells had a morphology consistent with expected stem cells, they were almost devoid of hemidesmosomes and rested upon a thin basement membrane. Moreover, these cells appeared to be in close proximity to limbal stromal cell extensions suggesting a possible route for crosstalk or direct cell-to-cell interaction (Fig. 2C and D). The micrograph shown in Figure 2F highlights a thin stromal cell extension presumably extending from the largest stromal cell observed in 2E, in direct connection to a small, basal epithelial cell at the edge of the crypt. Micrographs shown in Figure S1 clearly show basement membrane interruptions allowing direct contacts between stromal cell extensions and the basal epithelium.

### 3. Putative LESCs Make Direct Connections with Limbal Stromal Cells in the Stem Cell Niche

**Figure 3:**
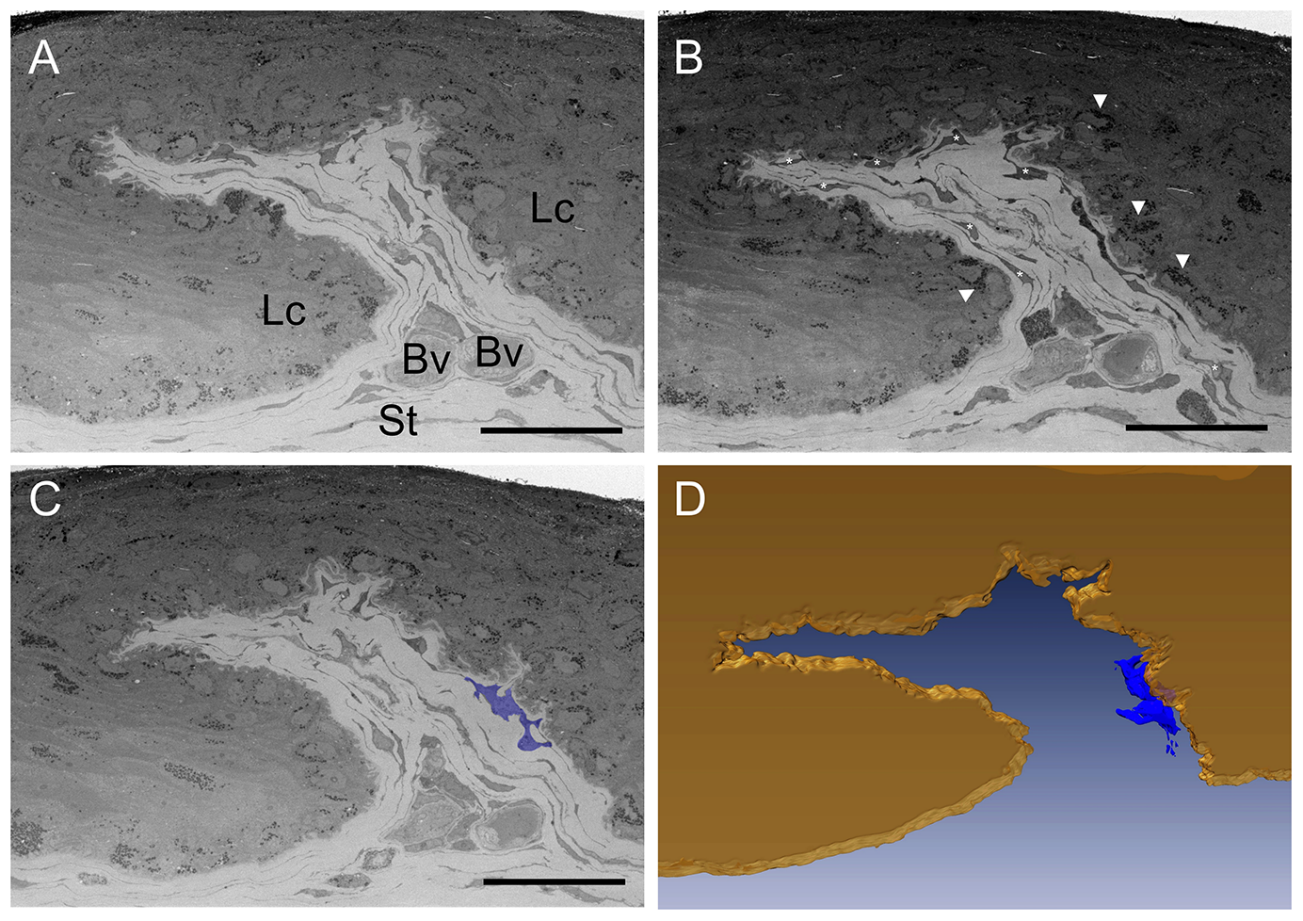
Limbal crypt ultrastructure observed by SBFSEM. Limbal crypt tangentially imaged through 70 μm from the corneal to the conjunctival side of the limbus. A, B and C represent non-sequential micrographs of the same 3D dataset. Manual segmentation followed by 3D reconstruction highlights the close proximity between the limbal epithelium (yellow volume in D) and a limbal stromal cell (white asterisks in B, blue area in C and blue volume in D) within the limbal crypt (Lc) suggesting a putative cell-to-cell contact. Lc: limbal crypt; Bv: blood vessel; St: Stroma. Arrowheads: melanocytes. Scale bars: 50 μm.

Low magnification imaging by SBFSEM directed at one limbal palisade separating two distinct LCs revealed the complexity of the stroma beneath the limbal epithelium (Fig. 3A-C and videos S1 and S2). Large and elongated stromal cells were closely associated with limbal basal epithelial cells (Fig. 3B). Within the epithelium, some cells contained electron dense cytoplasmic granules that had the potential to be melanosomes observed in limbal melanocytes. 3D reconstruction revealed the unusual proximity between limbal stromal and limbal basal epithelial cells although direct contact could not be confirmed at this magnification (Fig. 3C and D and videos 1 and 2).

**Figure 4:**
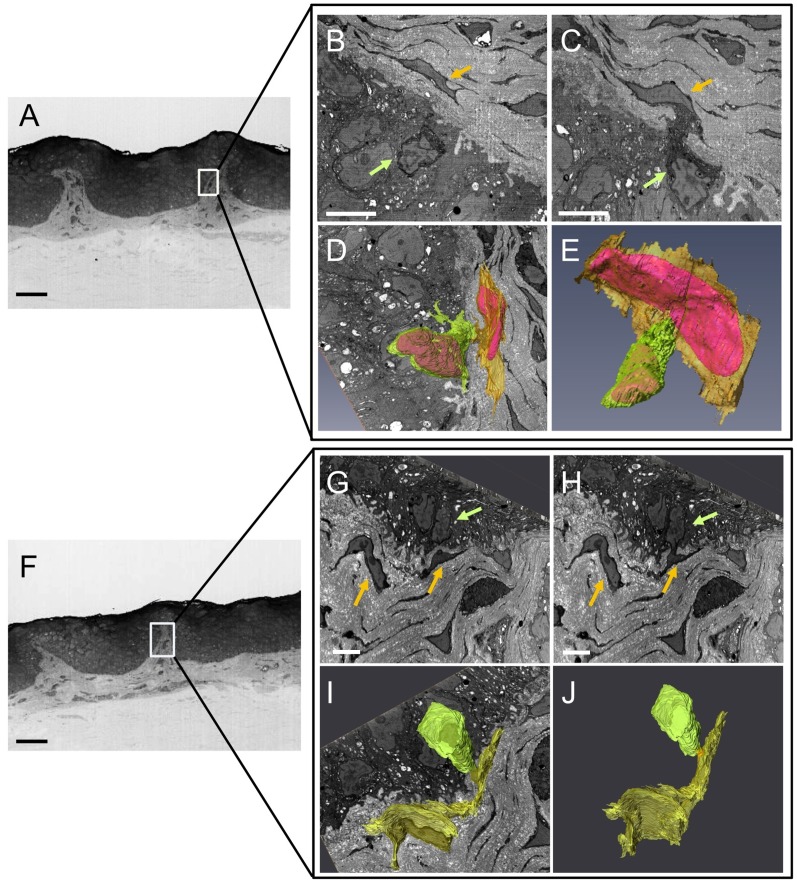
Cell-to-cell contacts imaged within the limbal crypts by SBFSEM. Box in A and F represents the area in which contact between putative LESC (green arrows in B, C, G, H and green volumes in D, E, I, J after 3D reconstruction) and limbal stromal cells (yellow arrows in B, C, G, H and yellow volumes in D, E, I, J after 3D reconstruction) was observed. Cell nuclei are represented in pink in D an E, the area of interaction between putative LESC and the limbal stromal cell is represented in orange (J). Scale bars: 50 μm (A, F); 5 μm (B, C, G, H).

High-magnification images of the interface between the basal epithelium and the limbal stroma on the edge of the LC (boxed area Fig. 4A and 4F) again showed the proximity between limbal stromal cells and putative LESCs (Fig. 4B and 4G). Figures 4C and 4H show that limbal stromal cells and putative LESCs previously observed on Figures 4B and G show a focal connection deeper in the stem cell niche. 3D reconstruction at this magnification revealed the morphological stem cell characteristics of the putative LESC and confirmed a direct interaction with a large and elongated stromal cell (Fig. 4D, E and 4I, J) at the edge of the limbal crypt.

### 4. Limbal Stromal Cells Expressing Mesenchymal Stem Cell Markers are More Frequently Observed within the Limbal Crypts

**Figure 5:**
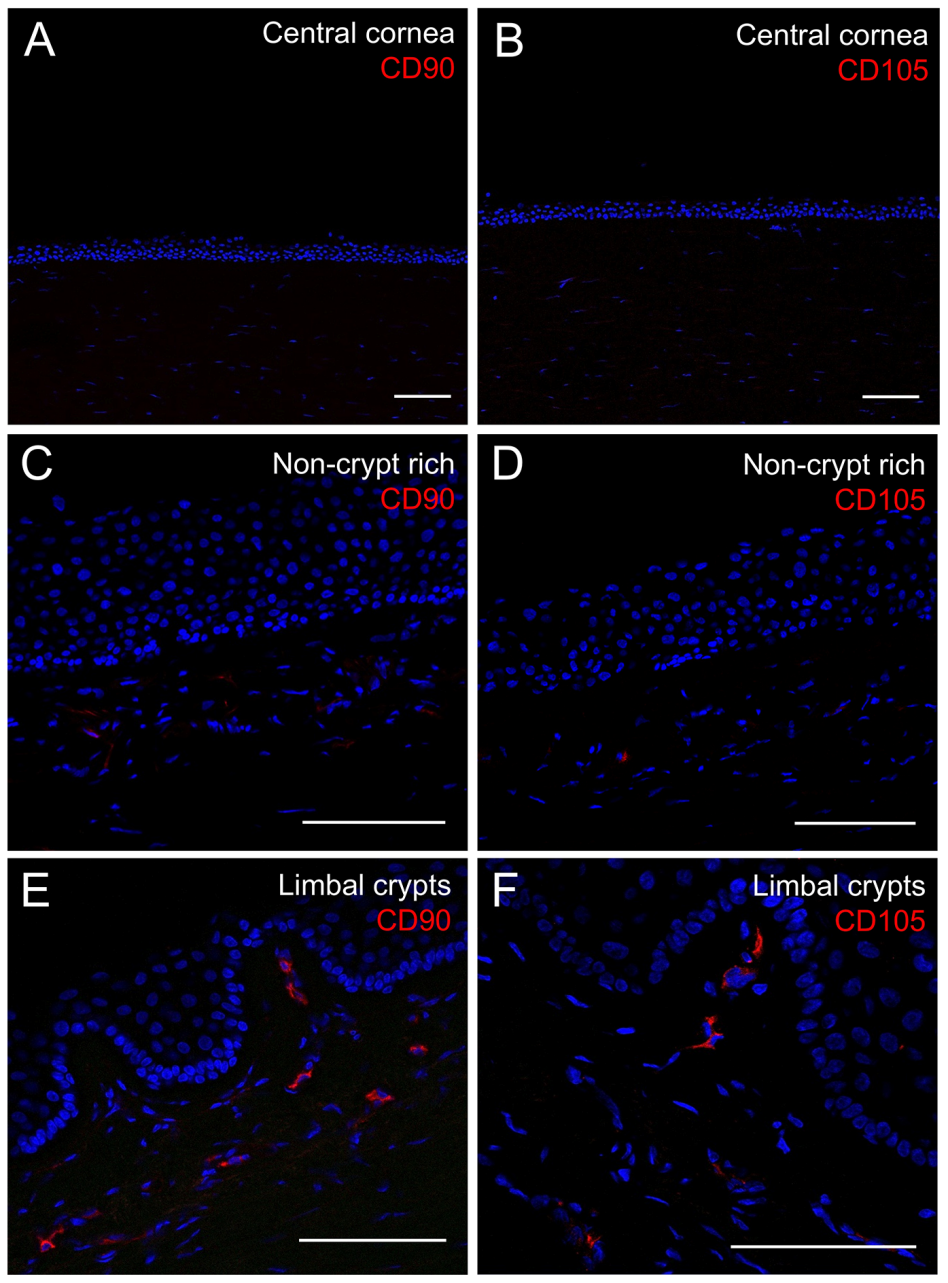
Results of immunohistochemistry staining for limbal mesenchymal cell markers CD90 and CD105 within the central cornea, the non-crypt rich limbus and the limbal crypts. Immunofluorescence suggests CD90 and CD105 expression is markedly increased by limbal stromal cells underlying the limbal crypts (E, F) compared to the non-crypt rich limbus (C, D). Central corneal sections were used as a negative control (A, B). Sections were counterstained with DAPI. Scale bars: 50 μm.

To determine whether limbal stromal cells involved in the direct contact with putative LESCs were limbal mesenchymal cells, we compared the expression of two mesenchymal stem cell (MSC) markers CD90 and CD105 in the central cornea, the non-crypt rich limbus and the limbal crypt-rich limbus. Immunostaining for both CD90 and CD105 was negative for both MSC markers in the central cornea (Fig. 5A and 5B). However, a sub-population of limbal stromal cells in the limbus expressed both CD90 and CD105 mesenchymal markers. Interestingly, the distribution of these limbal mesenchymal cells was not uniform. In the non-crypt rich limbus a small population of stromal cells expressed CD105 and weakly expressed CD90 (Fig. 5C and 5D). On the other hand, in crypt rich regions, there was a sub-population of limbal stromal cells beneath the LCs that were highly positive for either CD90 or CD105 MSC markers (Fig. 5E and 5F).

### 5. Limbal Melanocytes Interact with Putative LESC within the Limbal Crypts

**Figure 6:**
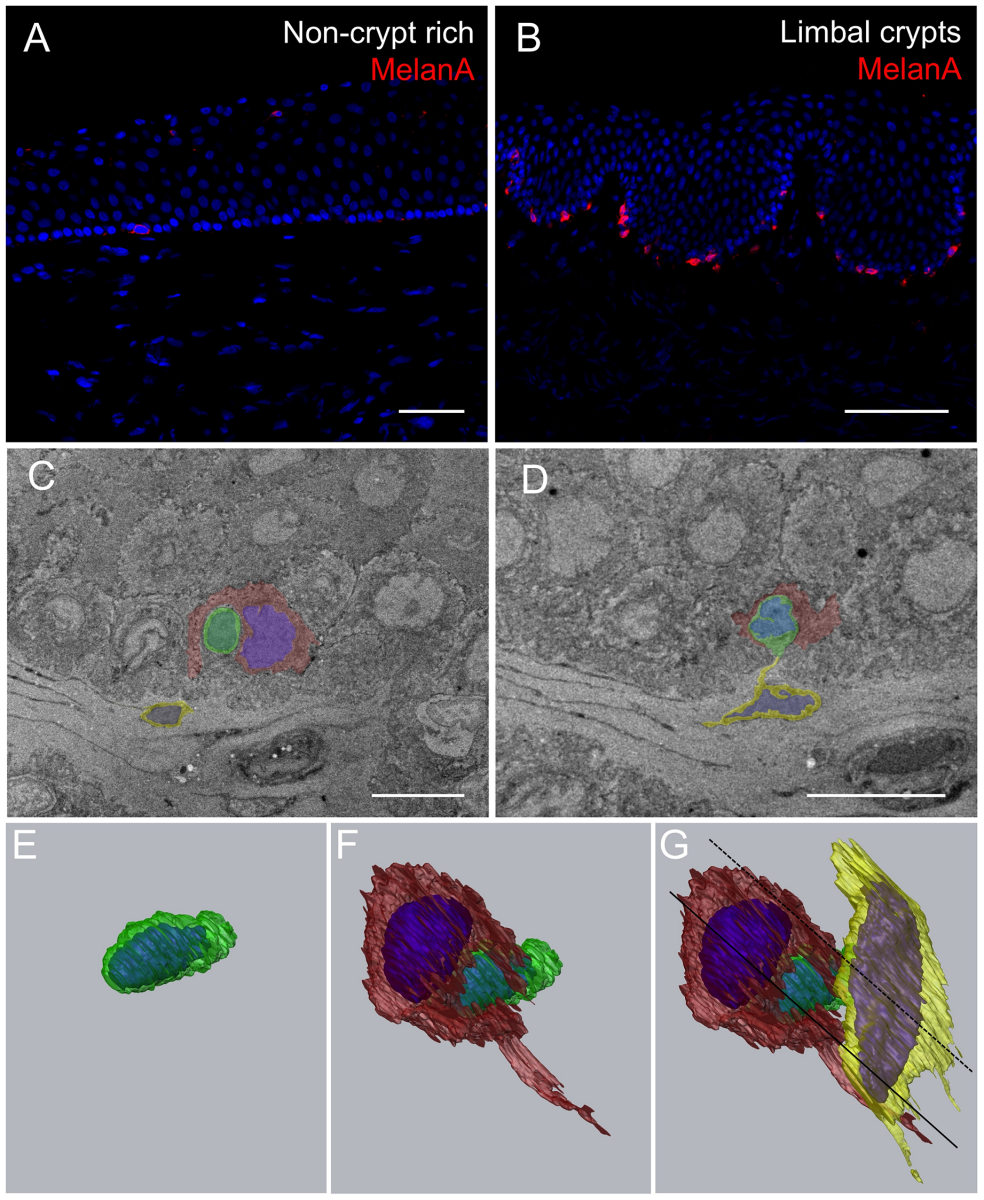
Melanocytes interact with putative LESCs in their niche. Immunohistochemistry reveals much more MelanA positive cells within the LCs than within the non-crypt rich limbal areas (A, B). Serial-block face scanning electron micrographs showing the edge of one limbal crypt (C, D). After manual segmentation and 3D reconstruction (E, F, G) putative LESC (smallest epithelial cell with a high nucleus/cytoplasm ratio) is represented in green, melanocyte (dendritic cell containing electron dense granules) in red, limbal stromal cell in yellow. Blue volumes correspond to nuclei. Continuous line in G corresponds to block face represented in C. Dashed line in G corresponds to block face represented in D. Scale bars: 50 μm (A, B) and 7 μm (C, D).

Immunostaining for MelanA, specifically expressed by melanocytes, identified a population of these pigmented cells within the epithelial basal layer of the limbal crypts where LESCs are concentrated. As shown in Figure 6A, limbal melanocytes were also observed, at a lower density, within the non-crypt rich limbus where they appeared dispersed between the epithelial layers. SBFSEM targeting of the edge of the limbal crypt (Fig. 6C and 6D) revealed that pigmented dendritic cells, corresponding to the morphology of limbal melanocytes, were closely associated with the smallest basal limbal epithelial cell which is in turn were located in close proximity to limbal stromal cells. After 3D reconstruction putative LESCs were found to directly connect with at least two non-epithelial cells (Fig. 6E). The apical aspect of the putative LESC connected with a dendritic limbal melanocyte (Fig. 6F) while the basal aspect connected with a limbal stromal cell (Fig. 6G).

## Discussion

The first indication of the presence of stem cells in the limbus was the observation in guinea-pig of centripetal migration of pigmented epithelial cells from the limbus following debridement of an area of adjacent epithelium [3]. Following DNA labeling, slow cycling epithelial cells were observed within the limbus of the mouse cornea, and these cells proliferated after injury [4]. Further *in vitro* clonal analysis demonstrated that the human limbus contained a population of limbal epithelial stem cells (LESCs) and that these cells could be used to generate a cohesive corneal epithelial sheets suitable for the treatment of ocular surface disease [12]. The assumption that there was a uniform distribution of LESCs around the peripheral cornea has been recently challenged as distinct anatomical features have been identified within the human limbus. These structures (including vascularized focal stromal projections also called ‘basal crypts’ [26], limbal crypts [19] and limbal epithelial crypts [17]) have been proposed as putative LESC niches. LCs, that correspond to downward projections of the limbal epithelium into the limbal stroma, are easily identified in normal eyes at low magnification within the limbal palisades of Vogt [19]. Clinically, LCs and focal stromal projections that are preferentially located at the superior and inferior meridians, could not be detected in patients affected by LESC deficiency [19].

In the present study we have used clonal analysis to compare the growth potential of LECs isolated specifically from crypt-rich and non-crypt rich limbal biopsies. We have demonstrated that biopsies taken from areas with limbal crypts contain a high population of limbal epithelial cells with the properties of stem cells [20]. Specifically, the generation of a greater number of holoclones in culture when compared to limbal epithelial cells isolated from limbal biopsies devoid of those structures. The generation of holoclone could also be observed when cells were isolated from the non-crypt rich limbus suggesting LESC are also present at a lower density outside the LCs. This ability to generate holoclones was indeed dramatically decreased when LECs were isolated from the non crypt-rich limbus hence confirming the LCs as a stem cell reservoir. We observed by transmission electron microscopy that the limbal crypts contain a sub-population of small, basal epithelial cells, a morphology characteristic of stem cells, [5] which were closely associated with the underlying limbal stromal cells suggesting the possibility of cell-to-cell contact. Cell-to-cell interaction between LESCs and their surrounding niche cells have been investigated previously. Higa et al. 2009, proposed that interaction between LESCs and 3T3 feeder cells mediated by N-cadherin is essential for the maintenance of the stem phenotype *in vitro*. However, such an interaction between putative LESCs and limbal stromal cells was not clearly identified in the native niche [23], [27]. On the other hand, Chen et al, observed that digestion of human limbal biopsies by collagenase released clusters of p63 positive (^+^ve), small epithelial cells closely associated with stromal vimentin^+^ve cells, which exhibited clonal growth in the absence of serum [24]. Interestingly, they demonstrated that association of LECs to their subjacent mesenchymal cells was essential for promoting clonal growth *in vitro.* Their observations support the concept of a direct crosstalk between LESC and limbal stromal cells for the maintenance of cell stemness in the native niche. In order to confirm our previous TEM observations we used SBFSEM. This allowed imaging in x, y and z directions and reconstruction of the 3D structure of the putative limbal stem cell [28]. SBFSEM imaging of the limbal basal epithelial layer revealed a clear and focal connection between small basal epithelial cells and their underlying elongated limbal stromal cells in the native niche. Such interaction was only observed within the limbal crypt and is facilitated by the presence of interruptions in the basement membrane that have been identified previously [29], acting like windows allowing cells from two opposing tissues to connect and interact with each other.

Small populations of mesenchymal stem cells have previously been observed in the human limbus. These limbal mesenchymal cells have the ability to grow clonally and to differentiate into various cell lineages under specific culture conditions, and it has been proposed that they maintain the phenotype of LESCs in the niche [30]–[34]. In the present study we investigated whether limbal mesenchymal cells were involved in the interaction with putative LESCs that we revealed by SBFSEM. Interestingly, we observed a population of limbal stromal cells positive for CD90 and CD105 mesenchymal stem cell markers that were more often present adjacently or beneath the limbal crypts where we demonstrated the highest population of LESCs to be located. However, at this stage, involvement of these limbal mesenchymal cells in the direct contact with putative LESCs that we observed by electron microscopy remains uncertain and would require further investigation. Higa et al. have indeed recently observed aquaporin1^+^ve elongated limbal stromal cells immediately located beneath the basement membrane in the proximity of N-cadherin^+^ve epithelial clusters that were also CK15^+^ve and p63^+^ve [27]. These aquaporin1^+^ve stromal cells appear to have the same location and morphology as the cells we have observed connecting the putative LESC, whereas CD90^+^ve and CD105^+^ve mesenchymal cells appear to lie deeper in the limbal stroma.

Limbal melanocytes have been observed to align with the basal layer of the epithelium and have been proposed to protect LESCs from damaging ultraviolet light [35]. In their study, Hayashi et al. 2007, observed that limbal melanocytes were also N-cadherin^+^ve and proposed that N-cadherin is involved in homotypic interactions with LESCs thus acting as associated cells maintaining LESC in the niche [10]. In the present study we observed that MelanA^+^ve limbal melanocytes were preferentially located within the basal epithelial layer of the limbal crypts where the LESCs are concentrated.

We propose a 3D model of the limbal stem cell niche in which the smallest basal epithelial cells within the limbal crypt are apically closely associated with pigmented limbal melanocytes and basally with limbal stromal cells. Such cell-cell interactions were not observed in the regions of the limbus normally devoid of limbal crypts. Despite the impressive resolution reached by volume electron microscopy, that allowed us to observe the putative LESC in their complex native microenvironment, we were unable to confirm the functional involvement of N-cadherin in this triple cell-to-cell interaction. This requires further investigation.

## Conclusion

In the present study, we have confirmed by *in vitro* clonal analysis that the previously identified limbal crypts provide a niche for the resident limbal epithelial stem cells. These observations directly support and strengthen the concept of targeted limbal biopsies for successful limbal stem cell therapy. High-resolution microscopy revealed clusters of small basal epithelial cells specifically observed within the limbal crypt. Volume electron microscopy revealed that the smallest basal epithelial cells with morphological stem cell characteristics were directly connected to the underlying limbal stromal cells via local basement membrane interruptions. After observing a higher population of limbal melanocytes within the limbal crypts and limbal mesenchymal cells underlying the crypts, we finally illustrated the first 3D reconstruction at a cellular-scale of the limbal epithelial stem cell niche.

## Supporting Information

Figure S1 **Transmission electron micrographs highlighting cell-to-cell contacts (black arrowheads) and basement membrane interruptions within the limbal crypts.** St: Stroma; Ep: Epithelium; Bm: Basement membrane. Scale bars 500 nm (A); 1 μm (B). Black arrowhead: Cell-to-cell contact between epithelial and stromal cell.
(TIFF)

Video S1 **Low magnification movie of serial-block face SEM dataset highlighting the complexity of the human limbal stroma within the limbal crypts.** Every image corresponds to a freshly exposed surface of the resin block after a 100 nm ultrathin section removal.
(MPG)

Video S2 **Medium magnification movie of serial-block face SEM dataset focused on the edge of one limbal crypt.** Note the cluster of small basal epithelial cells and the proximity of the underlying limbal stromal cells.
(MOV)
