# Supplementary figures and images for: Localisation of Epithelial Cells Capable of Holoclone Formation In Vitro and Direct Interaction with Stromal Cells in the Native Human Limbal Crypt
Source: PLoS One. 2014 Apr 8;9(4):e94283. doi: 10.1371/journal.pone.0094283 (PMC3979808; doi:10.1371/journal.pone.0094283)

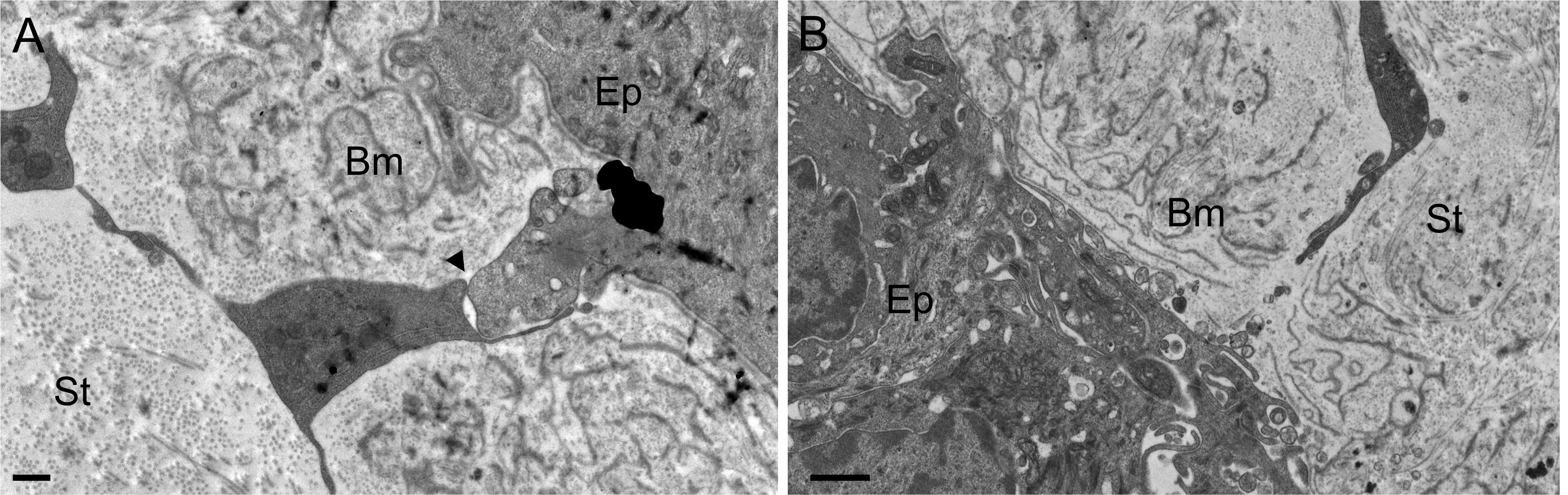

Supplement: Figure S1 — Transmission electron micrographs highlighting cell-to-cell contacts (black arrowheads) and basement membrane interruptions within the limbal crypts. St: Stroma; Ep: Epithelium; Bm: Basement membrane. Scale bars 500 nm (A); 1 μm (B). Black arrowhead: Cell-to-cell contact between epithelial and stromal cell. (TIFF) [file pone.0094283.s001.tiff]
